# Evolutionary ecology of intraspecific brain size variation: a review

**DOI:** 10.1002/ece3.627

**Published:** 2013-06-26

**Authors:** Abigél Gonda, Gábor Herczeg, Juha Merilä

**Affiliations:** 1 Ecological Genetics Research UnitDepartment of Biosciences, University of HelsinkiP.O. Box 65, FI-00014, Helsinki, Finland; 2 Behavioural Ecology GroupDepartment of Systematic Zoology and Ecology, Eötvös Loránd UniversityPázmány Péter sétány 1/C, H-1117, Budapest, Hungary

**Keywords:** Brain plasticity, brain size, evolution, natural selection, neural architecture, population differentiation

## Abstract

The brain is a trait of central importance for organismal performance and fitness. To date, evolutionary studies of brain size variation have mainly utilized comparative methods applied at the level of species or higher taxa. However, these studies suffer from the difficulty of separating causality from correlation. In the other extreme, studies of brain plasticity have focused mainly on within-population patterns. Between these extremes lie interpopulational studies, focusing on brain size variation among populations of the same species that occupy different habitats or selective regimes. These studies form a rapidly growing field of investigations which can help us to understand brain evolution by providing a test bed for ideas born out of interspecific studies, as well as aid in uncovering the relative importance of genetic and environmental factors shaping variation in brain size and architecture. Aside from providing the first in depth review of published intraspecific studies of brain size variation, we discuss the prospects embedded with interpopulational studies of brain size variation. In particular, the following topics are identified as deserving further attention: (i) studies focusing on disentangling the contributions of genes, environment, and their interactions on brain variation within and among populations, (ii) studies applying quantitative genetic tools to evaluate the relative importance of genetic and environmental factors on brain features at different ontogenetic stages, (iii) apart from utilizing simple gross estimates of brain size, future studies could benefit from use of neuroanatomical, neurohistological, and/or molecular methods in characterizing variation in brain size and architecture.

Evolution of brain size and architecture is a widely studied topic. However, the majority of studies are interspecific and comparative. Here we summarize the recently growing body of intraspecific studies based on population comparisons and outline the future potential in this approach.

## Introduction

The brain has always been of interest to almost every field of biology dealing with animals due to its role in shaping the outcome of almost any contact between an individual organism, and its environment. One of the simplest, yet often used, proxies for the brain's evolutionary state of development is its size (Striedter 155). Even though the significance of the overall brain size – or even the size of the main brain parts (depending on the taxon) – and what exactly they tell us about the individual or species intelligence and cleverness is debated (Healy and Rowe 58; Chittka and Niven 17), overall brain size is still used (in cases where there is no better substitute) as a proxy of intelligence and cognitive ability (Gibson 42; Striedter 155). Even methods estimating brain size indirectly are in use and advancing recently (e.g., Logan and Clutton-Brock 88; Soul et al. 153). There are a number of potential variables to analyze and methods to measure those variables regarding brain size, but considering the available reviews on this topic (e.g., Striedter 155; Deaner et al. 21; Healy and Rowe 58; Dechmann and Safi 22) we do not discuss this topic further.

Energetic constraints, stemming from the fact that the brain tissue is extremely expensive to maintain (Aiello and Wheeler 2; see also Navaterre et al. 103; Allen and Kay 6; Warren and Iglesias 165; Kotrschal et al. 165), should impose strong selective pressure against nonadaptive variability and changes. Hence, an increase in brain size can happen only when the benefits of a larger brain outweigh the cost of production and maintenance. For example, selection for increased cognitive ability should favor increased brain size, but only when enough resources can be secured to cover the increased energetic needs without loss in other aspects of fitness. For the same energetic reason as above, the size of a given brain part might be a good indicator of its importance, and reflect the way the given species or population has adapted to its environment and prevailing selective regime (Krebs et al. 78; de Winter and Oxnard 171; Gonzalez-Voyer and Kolm 48).

Enormous variation in brain size – both in absolute and relative terms – has been reported in a number of taxa (e.g., mammals: Harvey et al. 56; fish: Kotrschal et al. 74; birds: Day et al. 20). Our current knowledge about variation in brain size and architecture in the wild is based on two main lines of research. First, on interspecific comparative studies focusing on relationships between brain size and environmental parameters as well as between brain size and behavior and/or life history trait variation (e.g., food hoarding: Garamszegi and Eens 38; social complexity: Dunbar and Shultz 30,b; environmental complexity: Pollen et al. 119; parental care type and pair bonding: Gonzalez-Voyer et al. 49). Second, on studies of adaptive phenotypic plasticity in brain size (reviewed in: van Praag et al. 121; Mohammed et al. 98).

However, to fully understand the evolution of a quantitative trait, one should (i) establish the individual phenotypic variation in the trait in question, (ii) estimate selection acting on the different phenotypes, (iii) estimate the heritability of the trait, and ultimately, (iv) understand its genetic underpinnings. Unfortunately, none of these can be addressed by the above mentioned interspecific comparative evolutionary studies (and obviously not by intrapopulation phenotypic plasticity studies). To achive these goals, intraspecific evolutionary studies are needed accompanied by phenotypic plasticity studies. The aim of the present paper is to bring attention to the importance of applying intraspecific evolutionary approaches to understand brain evolution.

We will first briefly summarize what is known about variation in brain size and architecture (defined as the size of different brain parts in comparison to each other, to the total brain, and to body size) thanks to the interspecific comparative studies and research on adaptive phenotypic plasticity. Second, we introduce the emerging field of intraspecific brain evolution focusing on interpopulation variation in brain size and size of brain parts, as well as on the interpopulation variation in the plasticity of these traits. Finally, we outline future avenues for studies aimed to increase our understanding of brain evolution and factors driving it.

## Macroevolution and Comparative Studies – Comparing Taxa

**Figure 1:**
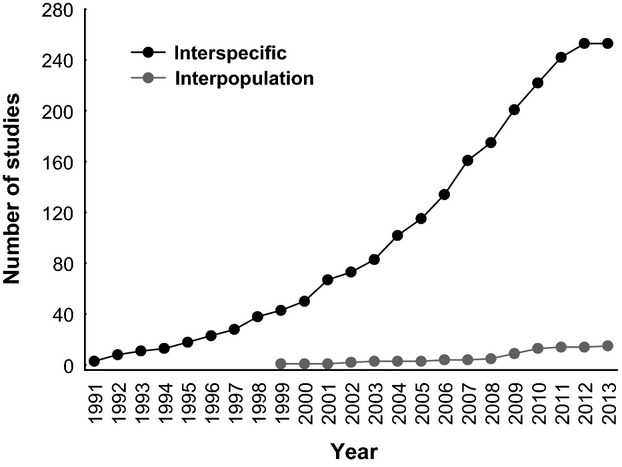
Cumulative number of evolutionary studies focussing on variation in brain size and architecture by comparing species or higher taxa (“Interspecific”) versus comparing populations of a single species (“Interpopulation”). Data are based on a literature search in Web of Science, using the search terms: “brain size” and “evolution”. The situation is depicted until the end of April, 2013.

A large body of macroevolutionary research has been conducted on different taxa in attempts to understand the major evolutionary forces behind brain size evolution (e.g., Clutton-Brock and Harvey 18; Kotrschal et al. 74; Striedter 155; Shumway 143; Weisbecker and Goswmai 166; Fig. 1). Giving a full overview on this topic is outside of the scope of this treatment (see Healy and Rowe 58 for a summary). However, we will briefly review the main findings and the proposed selective forces that shape the evolution of brain size and architecture, as they provide templates for further interpopulation comparisons and form a basis for comparing macroevolutionary and microevolutionary patterns. Correlations have been revealed between brain size or size of different brain structures and different environmental factors (e.g., Pollen et al. 119), seasonality (van Woerden et al. 172), life history (e.g., Gonzalez-Voyer et al. 49; Isler 66; Barton and Capellini 8), intensity of sexual selection (Fitzpatrick et al. 36), behavioral (Ratcliffe et al. 125; Aviles and Garamszegi 7), and morphological traits (gut size: Aiello and Wheeler 2; testis size: Pitnick et al. 118; body size: Gonzalez-Voyer et al. 50) on interspecific (or higher) level after controlling for phylogenetic nonindependence. However, most of these studies are done on primates and birds. Specifically, the evolution of the exceptionally large relative brain size of primates (and especially humans) has mainly been studied in light of sociality (e.g., Dunbar and Shultz 30,b). Social complexity, requiring life in large and complex groups or in pair bonds is accepted as the main driver of primate, especially human, brain size evolution (also known as “social brain hypothesis”, e.g., Dunbar 28; Dunbar and Shultz 30,b; Perez-Barberia et al. 115). Apart from the increase in overall brain size, the size of the neocortex and hippocampus has received special attention. This is because the neocortex in primates (and especially in humans) has increased disproportionally during its evolution, and the hippocampus plays an important role in memory and learning, which have always been of human interest (Striedter 155). In the case of birds, most of the focus has been on brain size or size of the forebrain, especially the telencephalon and the hippocampus, for the same reason as in primates. The main correlates and suggested drivers behind the evolution of these neural structures are suggested to be selection forces stemming from migration and foraging innovation (e.g., Lefebvre et al. 80; Sol et al. 149,b).

Even though comparative studies form the cornerstone of our current knowledge about brain size evolution, they are by nature correlative and therefore causations are hard to prove with the approaches used.

## Phenotypic Plasticity in Brain Size – Comparing Individuals

Besides local adaptation driven by selection on heritable phenotypic variation resulting in adaptive genetic divergence, also adaptive phenotypic plasticity (e.g., West-Eberhard 169) can allow adaptive adjustment or acclimation to prevailing environmental conditions (e.g., Ghalambor et al. 41). Studies on brain development have demonstrated that those parts of the brain that are likely to be important in a particular context develop more than those of less importance in that context (Kihslinger and Nevitt 70; Kihslinger et al. 71; Lisney et al. 86). Again, as the brain is an expensive tissue to develop and maintain (Aiello and Wheeler 2; Kotrschal et al. 77), energetic constraints should impose strong selection against nonadaptive modifications of brain. Hence, phenotypic plasticity in the brain can be expected to have an adaptive value.

Plastic changes in brain size occur in nature. For instance, there is strong evidence for seasonal plasticity in the size of certain neural structures (e.g., in the song control center of songbirds: Nottebohm 106; Tramontin and Brenowitz 159), in the anatomy of the human hypothalamus and hippocampus (Hofman and Swaab 62), in the volume of hypothalamic nuclei in humans (Hofman and Swaab 61), and in the hippocampal morphology of the white-footed mouse *Peromyscus leucopus* (Pyter et al. 124). Mental and physical training also appear to influence neural architecture (e.g., Patel et al. 113; Gould et al. 51,b; van Praag et al. 121; Brown et al. 12; Rhode et al. 129; Draganski and May 26). For instance, the size of the posterior hippocampus of London cab drivers increases with time spent as a cab driver (Maguire et al. 92). Additionally, hippocampus-dependent learning has been shown to increase the number of newly generated cells of the hippocampus in rats (Gould et al. 51,b), spatial learning induced neurogenesis in the hippocampus of birds (Patel et al. 113), and voluntary running resulted in enhanced neurogenesis in the hippocampus of adult mice (van Praag et al. 120; Brown et al. 12; Rhode et al. 129). Change in social status altered the size of song control centers of songbirds (Voigt et al. 162) and the size of somatostatin-containing neurons in fish (Hofmann and Fernald 63), while social rank has been found to correlate with forebrain cell proliferation rate in fish (Sørensen et al. 152). Further, the size of brain parts that are of importance in certain life stages can also change reversibly. For example, shifts in habitat, diet, or behavior can alter the relative size of the main sensory brain areas in fish (Wagner 163; Lisney et al. 86), while changes in the size of different brain parts during pregnancy in women is likely to reflect the different need for the function that given brain part is responsible for (Oatridge et al. 107).

Besides naturally occurring plastic changes, brain plasticity can be induced experimentally as well. Such experimental studies have shed light on the effects of abiotic and biotic environmental complexity on brain development (reviewed in: van Praag et al. 121; Mohammed et al. 98). Some of the main studies are compiled in Table 1. For example, rodents exposed to enriched (stimulus rich) abiotic environments had increased brain size (Diamond et al. 25; Rosenzweig and Bennett 131), more hippocampal neurons (Kempermann et al. 69), and elevated level of neurogenesis (Kempermann et al. 69; Nilsson et al. 104) compared to those living in stimulus poor environments.

**Table 1:** Experimental studies on brain plasticity investigating the effects of different abiotic and biotic environmental factors

Environment	Factor	Affected brain region	Species	References
Abiotic	Enriched environment	Brain size	Norway rat, *Rattus norvegicus*	Diamond et al. (25), Rosenzweig and Bennett (131)
		Hippocampal neurons	House mouse, *Mus muscuslus*	Kempermann et al. (69)
		Neurogenesis	House mouse, *Mus musculus*; Norway rat, *Rattus norvegicus*	Kempermann et al. (69), Nilsson et al. (104)
		Cell proliferation in the telencephalon	Coho salmon, *Oncorhynchus kisutch*	Lema et al. (84)
		Size of the cerebellum	Steelhead trout, *Oncorhynchus mykiss*	Kihslinger and Nevitt (70)
	Captive rearing	Brain size, size of the optic tectum and telencephalon	Guppy, *Poecilia reticulata*	Burns and Rodd (13), Burns et al. (14)
		Size of the olfactory bulb and telencephalon	Chinook salmon, *Oncorhynchus tshawytscha*	Kihslinger et al. (71)
		Size of several brain parts, (in some case) the size of the overall brain	Nine-spined stickleback *Pungitius pungitius*	Gonda et al. (46)
		Telencephalon	Three-spined stickleback (*Gasterosteus aculeatus*)	Park et al. (112)
	Training	Hippocampus	Human, *Homo sapiens*	Maguire et al. (92)
		Several brain areas and activities	Human, *Homo sapiens*	Draganski and May (26)
Biotic	Social environment	Optic tectum, bulbus olfactorius	Nine-spined stickleback, *Pungitius pungitius*	Gonda et al. (43)
		Sensory brain areas	Common frog *Rana temporaria*	Gonda et al. (45), Trokovic et al. (160)
		Number of new neurons in the dentate gyrus	Prairie vole, *Microtus ochrogaster*	Fowler et al. (37)
		Neuronal recruitment	Zebra finch, *Taeniopygia guttata*	Lipkind et al. (85), Adar et al. (1)
		Size of the brain and the proportion of different brain areas	Desert locusts *Schistocerca gregaria*	Ott and Rogers (108)
		Overall brain size, optic tectum	Guppy, *Poecilia reticulata*	Kotrschal et al. (75,b)
	Predation pressure	Olfactory bulb, hypothalamus	Nine-spined stickleback, *Pungitius pungitius*	Gonda et al. (46)
		Overall brain size	Common frog, *Rana temporaria*	Gonda et al. (45)

Studies on the effects of abiotic environmental factors are only a representative subset of studies, while all studies (to our knowledge) on the effects of biotic environment are listed.

Captive rearing has been shown to reduce brain size in guppies, *Poecilia reticulata* (Burns and Rodd 13; Burns et al. 14), size of the olfactory bulb and telencephalon in the Chinook salmon, *Oncorhynchus tshawytscha* (Kihslinger et al. 71) and guppies (Burns and Rodd 13), and the relative size of every main brain part as well as the size of the whole brain in nine-spined sticklebacks, *Pungitius pungitius*, from particular habitats (Gonda et al. 46; Table 1). Kihslinger and Nevitt (70) showed that adding only a single rock to the rearing tank can increase the size of the cerebellum of salmons at very early life stages, while changes in cell proliferation in the telencephalon (although without changes in the size of the given brain part) can be induced by environmental complexity in juvenile Coho salmon (Lema et al. 84). These latter studies are of a special importance, as they may have important implications to fish aquaculture and reintroduction programs. However, the effects of hatchery rearing are not always so simple and clear and can even differ between breeding lines (Kotrschal et al. 75, b).

Different biotic environmental factors have also been shown to influence brain development, but the number of studies on this effect is still far lower than those of the abiotic environment – all studies on the effects of biotic environment are listed in Table 1. Furthermore, many commonplace and ecologically important biotic interactions such as social environment, predation risk, or competition have rarely been investigated (but see, e.g.,: Gonda et al. 43, 45, 47; Trokovic et al. 160). It has been shown that social environment can alter brain development, especially the sensory brain areas, both in the nine-spined stickleback (Gonda et al. 43) and the common frog (*Rana temporaria;* Gonda et al. 45; Trokovic et al. 160). Individually reared fish developed smaller optic tectum and larger bulbus olfactorius than group reared fish, and in some highly aggressive populations group rearing resulted in decreased overall brain size (Gonda et al. 43). The development of the main sensory brain areas were also affected by density in both tadpoles and metamorphosed froglets (Gonda et al. 45; Trokovic et al. 160). Social isolation decreased the number of new neurons in the dentate gyrus of prairie voles (Fowler et al. 37), while social complexity increased neuronal recruitment in birds (Lipkind et al. 85; Adar et al. 1). The change in density between life phases of desert locusts alters the size of the brain and the proportion of different brain areas; solitarious locusts have smaller brains as compared to gregarious locusts (Ott and Rogers 108). In a recent study, Kotrschal et al. (75) demonstrated how sex ratio in the social environment induces sex-specific plasticity in total brain size and optic tectum size in guppies: male brains were smaller in same-sex than in mixed-sex groups, while female optic tecta were smaller in mixed-sex than in same-sex groups. Perceived predation risk resulted in decreased size of the olfactory bulb in some populations of nine-spined sticklebacks (Gonda et al. 47) while common frog tadpoles developed smaller brains under predation risk in low density (=high per capita predation risk) than in high density or in the absence of predator (Gonda et al. 45).

## Beyond Comparative Studies and Phenotypic Plasticity

The above detailed interspecific correlative studies form the basis of our present knowledge about how brain size/architecture evolved, and studies on phenotypic plasticity have highlighted the importance of ontogenetic variation in brain development. However, these pillars together are still far from providing a complete picture about the processes resulting in the observed brain variation in the wild. The proposed factors that might shape the brain both on evolutionary and ontogenetic scales are well established in most cases (e.g., Dunbar 28; Shumway 142, 143), but several critical questions remain unanswered. Are the present environmental factors imposing selective pressures on the brain the same as the ones that originally lead to the present forms? What is the heritability of brain size and how is it influenced by environmental variability? Likewise, what is the relative importance of phenotypic plasticity versus local adaptation in explaining variation in brain size and architecture in the wild? In other words, to what extent is the variation we see among wild populations in brain architecture caused by differences in the genetic constitution of the population, rather than environmentally induced plasticity? Can brain plasticity itself be under selection and expressed differently in different populations? Within the genetically based patterns, what is the relative importance of natural selection versus drift in explaining the observed differentiation? Are brain size and architecture differences coded by a small number of genes with major effects, or rather by a large number of genes with small effects? Are there strong genetic correlations between the sizes of different brain parts, that is, strong constraints on evolution of brain architecture? What are the fitness consequences of individual variation in brain size?

The list could be continued, and it is clear that a number of fundamental evolutionary questions about brain variation simply cannot be answered by interspecific evolutionary or intrapopulation plasticity studies. To fill the gap between the two, and to answer most of the questions listed above, population comparisons within a single species – coupled with studies of within-population variation – are needed. In other words, evolutionary studies should be scaled down to the inter- or even intrapopulation level, while plasticity studies need to be scaled up to the interpopulation or even interspecific level to provide answers to the questions posed.

## Microevolutionary Studies – Comparing Populations

Macroevolutionary brain studies rely on the assumption that variation between species is much higher than variation within species. Even though extensive within species brain size variation has been reported (e.g., Kolm et al. 73; Møller 99; Gonda et al. 46), variation between species is indeed likely to be larger than that within species in most cases (Garamszegi and Eens 38; Garamszegi et al. 40). However, the intraspecific variation in brain size and architecture is still very informative and important for our understanding of evolutionary processes. Contrary to studies on the species level, evolutionary studies on brain size at the intraspecific level have only recently started to receive the attention of evolutionary biologists (e.g., Gonda et al. 44, 46; Kolm et al. 73; Roth and Pravosudov 132; Crispo and Chapman 19; Fig. 1; Table 2). As with all new research areas, the first studies are explorative and are paving the road for more in depth studies to come. In the case of evolutionary studies of brain size at the intraspecific level, early studies have used rather rough brain size measurements (e.g., Burns and Rodd 13) or even head volume as an indicator for brain size (Møller 99). Although these proxies of brain size are believed to be good estimates of intelligence and cognitive ability (see Introduction), more refined techniques (see “Future directions”) can improve the resolution and provide more fine-tuned analyses of specific hypothesizes to be tested. Perhaps more importantly, as compared to interspecific studies, intraspecific studies provide numerous conceptual advantages in testing hypotheses about the evolution of brain size and architecture.

**Table 2:** Synopsis of evolutionary studies of brain variability based on interpopulation comparisons

Taxon	Trait	Proposed correlates	Method	Sample	References
Human, *Homo sapiens*	Brain size	Intelligence quotient	Magnetic resonance imaging	“W”	Rushton and Ankney (135)
Marsh wrens *Cistothorus palustris*	Song control nuclei	Song learning, repertoire size	Histology	W	Canady et al. (15)
White-crowned sparrow, *Zonotrichia leucophrys*	Hippocampus size and neuron number	Migratory behavior	Histology	W	Pravosudov et al. (123)
Black-capped chickadee, *Poecile atricapillus*	Hippocampus size and neuron number	Latitude, temperature, snow cover, day length	Histology	W	Pravosudov and Clayton (122), Roth and Pravosudov (132), Roth et al. (134)
Dwarf Victoria mouthbreeder, *Pseudocrenilabrus multicolor victoriae*	Brain mass, plasticity	Oxygen level of water, dispersal potential	Weighing	CG	Crispo and Chapman (19), Chapman et al. (16)
Brown trout, *Salmo trutta*	Brain size and architecture	Mating strategy, sex	Volume calculation on photos	W	Kolm et al. (73)
Three-spined stickleback, *Gasterosteus aculeatus*	Brain size and architecture	Foraging strategy (limnetic, benthic), sex	Shape analysis on photos	W	Park and Bell (111)
Nine-spined stickleback, *Pungitius pungitius*	Brain size and architecture	Predation, environmental complexity	Volume calculation on photos	W & CG	Gonda et al. (46, 44)
Lake whitefish, *Coregonus clupeaformis*	Brain mass	Predation, prey community	Weighing	W	Evans et al. (33)
Honey bee, *Apis mellifera*	Total brain and mushroom body size	Learning performance	Histology	W	Gronenberg and Couvillon (53)
Small white*, Pieris rapae*	Total brain and mushroom body size	Learning	Histology	CG	Snell-Rood et al. (147)

“Proposed correlates” identifies the factor that might have contributed to the observed divergence in brain. “Sampling” tells whether the studies were done on wild caught animals (W) or on animals reared in controlled laboratory environment (common garden, CG). Note that we treated the *Gasterosteus aculeatus* and *Coregonus clupeaformis* studies (refs. Park and Bell (111), Evans et al. (33), respectively) as interpopulation studies, but the compared populations might also be seen as already distinct species.

Firstly, comparisons of brain size and architecture differences among populations of the same species inhabiting different selective environments could provide explicit means to differentiate between various microevolutionary processes, such as natural selection and genetic drift (e.g., Merilä and Crnokrak 96), as causes of observed differentiation. By comparing the levels of population differentiation in quantitative phenotypic traits (*Q*_ST_) with the degree of differentiation in neutral genetic markers (*F*_ST_), one can probe the causes of differentiation (e.g., Leinonen et al. 82). If *Q*_ST_ > *F*_ST_, the patterns/differences in the given phenotypic trait among population inhabiting different habitats are likely to reflect local adaptation (i.e., evolutionary divergence). If *Q*_ST_ = *F*_ST_, this indicates that the observed differences do not exceed what would be expected due to genetic drift alone. On the other hand, if the *Q*_ST_ < *F*_ST_, the examined populations have diverged less than expected by drift alone, and the populations are likely to be under similar selective pressures (Merilä and Crnokrak 96). Thus far, this approach has not been applied in any study of brain evolution, and hence, formal tests of adaptive differentiation are as yet lacking.

Apart from the *Q*_ST_−*F*_ST_ comparisons, there is another way to test for links between the phenotypic expression of a trait and selective forces shaping the phenotypic appearance of that trait: simple selection experiments, where a group of individuals is subjected to a selective force like predation and individual phenotype can be linked to fitness. Such experiments have been frequently employed to study the functional significance of phenotypic variation of different traits (e.g., Reznick and Ghalambor 128; Leinonen et al. 83). However, no study has as yet used this kind of experimental approach to verify the actual impact of a particular brain phenotype on individual performance or fitness. There is another reason why intraspecific comparative studies can be more informative and provide us with more detailed answers about the evolutionary forces behind brain size evolution than the otherwise undeniably important interspecific comparative studies. This resides in the fact that most populations are likely to be found in the selective environment that actually shaped their brains, while this is less likely to be the case in species comparisons. Hence, population comparisons can help us to identify the most important environmental factors selecting for size and structural changes in the brain, and by studying recently established populations/recent radiations, natural selection acting on the brain can be “caught in action”.

Based on interpopulation comparisons, environmental variables that might have contributed to the reported brain size/architecture divergence, as well as to correlated life history and/or behavioral traits, have been identified (Table 2). For example, in food hoarding animals, good memory (and hence the associated neural basis) is essential for survival, especially under harsh environmental conditions. Indeed, environmental harshness correlates with the size and neuron number of hippocampus in the black-capped chickadee (*Poecile atricapillus*; Pravosudov and Clayton 122; Roth and Pravosudov 132), even when one of the environmental factors of harshness (the day length) was controlled for (Roth et al. 134). In two other studies, a difference in the predatory regime was the main proposed factor behind brain architecture divergence in nine-spined sticklebacks (Gonda et al. 44, 46). Brain comparisons between populations and the main findings of those studies are summarized in Table 2.

Evolutionary brain studies that were based on comparisons of individuals of the same population, or several populations but neglect population origin, might be of less direct importance in the context of local adaptation. However, such studies (e.g., MacDoughall-Shackleton et al. 90; Møller 99; Wilson and McLaughlin 170) have identified interesting behavioral and life history traits which might be worth investigating on the interpopulation level. For example, the correlation between size of song control centers in the brain and song repertoire in songbirds has received much attention (e.g., Ward et al. 164; Airey and DeVoogd 3; Garamszegi and Eens 39), and sometimes yielded conflicting results (for review see Garamszegi and Eens 39). However, Canady et al. (15), studying marsh wrens (*Cistothorus palustris*) both in nature and in the lab, were among the first to show among-population variation in song brain centers. Also fish with different foraging behaviors differ in their brain architecture: actively foraging brook chars (*Salvelinus fontinalis*) have larger telencephala than their less active conspecifics (Wilson and McLaughlin 170). Different proxies of brain size (brain mass and head size) in the barn swallow (*Hirundo rustica*) were also shown to be in positive correlation with several factors, including migratory behavior, offspring defense, recapture probability (i.e., learning), sex, and social environment (Møller 99).

Some quantitative genetic work has already been done to study the heritability of brain size and architecture mainly in humans and primates. Differences in gross brain morphology were found to be heritable (*h*^*2*^ ≈ 0.66–0.97) on the basis of analyses utilizing known pedigrees or exploiting the possibilities in human twins (e.g., Hulshoff Pole et al. 65; Peper et al. 114). Likewise, heritabilities of brain size, cerebral volume, and gray matter volume in baboons, *Papio hamadryas*, were found to be high (*h*^*2*^ ≈ 0.67–0.86; Rogers et al. 130). Similar results have been found in zebra finches (*Taeniopygia guttata*), where brain weight and telencephalon volume were also highly heritable (*h*^*2*^ ≈ 0.49–0.63), and size of some song control nuclei had lower but still significant heritabilities (*h*^*2*^ ≈ 0.03–0.16) based on the application of “animal model” analyses on full-sib families (Airey et al. 4). These studies are promising, as they indicate high evolvability of different brain traits in distant taxa. Recent studies that have employed artificial selection either directly on brain (Kotrschal et al. 77) or on other traits (Kolb et al. 72) also strengthen the view that brain size and structures are highly evolvable. At the same time, they raise interesting questions from the evolutionary point of view: if the variation in the brain size and size of different brain parts has important consequences on fitness, how are we to explain these high heritabilities? Namely, traits with close association to fitness are expected to have low heritabilites (Mousseau and Roff 102; Merilä and Sheldon 97). Given the functional importance and the energetic constraints of maintaining brain tissue, it is intriguing that the heritabilites of brain size traits appear to be this high.

We see many possibilities in quantitative genetic studies of brain size variation, especially in species where large-scale breeding experiments are possible. As compared to studies of primates and humans, in which experimental work is difficult and logistically constrained, organisms with shorter generation times – such as small-sized fish and possibly some amphibians – might provide promising models for quantitative genetic work. However, whichever species one chooses to utilize, one of the limiting factors in studies of brain variability resides is obtaining high-resolution data on brain size variation. Hence, as Houle et al. (64) recently pointed out, high-throughput phenotyping methods need to be developed to meet the demand of measuring hundreds (preferably thousands) of brains.

Taken together, intraspecific studies on brain variation have started to accumulate (Fig. 1). These studies suggest that there is a great deal of variation in brain phenotypes both among and within populations, as well as covariation between brain phenotypes and environmental (and behavioral or life history traits) variables within a single species. Furthermore, the quantitative genetic studies thus far indicate high heritability of brain size and the size of different brain parts, which together with the functional – and therefore also evolutionary – significance of brain variation suggest ample opportunity for local adaptation in brain traits. However, the evidence for local adaptation in brain size and architecture from the wild is still scant. While some of the studies have utilized common garden approaches, most of the studies have relied on wild caught animals and the genetic – and hence – adaptive basis of the observed differentiation remains questionable (e.g., Gonda et al. 46).

## Brain Plasticity From an Evolutionary Perspective – Comparing Populations

As highlighted in our introduction, phenotypic plasticity in brain size has been demonstrated several times. It is still debated if phenotypic plasticity itself is an evolvable trait or just the first step toward adaptation in general (West-Eberhard 169; DeWitt and Scheiner 24; de Jong 68; Pigliucci et al. 117; Pfennig et al. 116; Snell-Rood 146). Work done on brain plasticity so far is not placed to challenge any of these views. Contrary to the large amount of brain plasticity studies done at the within-population level, we are aware of only three studies investigating the evolution of brain plasticity. Nine-spined sticklebacks showed habitat-dependent population divergence in brain plasticity induced by sociality (Gonda et al. 43): pond sticklebacks (which are the only fish species in their ecosystems) developed relatively smaller brains in groups than in isolation, while marine sticklebacks (which are members of a diverse fish fauna with numerous predators in their ecosystems) showed an opposite trend. It was suggested that under heavy piscine predation, marine sticklebacks developed some mechanisms that eliminate the social stress stemming from aggressive encounters. Further, another study showed that nine-spined sticklebacks from pond environment increased the size of their bulbi olfactorii in the presence of predation pressure while this brain part remained the same in marine fish, however, marine fish in general developed larger brain than pond fish (Gonda et al. 47). The results suggest that predation pressure increase the size of the olfactory brain center both on evolutionary and ontogenetic scales. A third study showed that African cichlids (*Pseudocrenilabrus multicolor victoriae*) with higher dispersal potential have more plastic (and also smaller) brains than their conspecifics without high dispersal potential (Crispo and Chapman 19). Finally, though not directly addressing the question of population variation in brain plasticity, it has been found that the effect of captive rearing can be habitat specific in nine-spined sticklebacks, whereas pond fish developed smaller brains in captivity than in the wild, while marine fish developed similar sized brains both in the wild and in the lab (Gonda et al. 46).

Based on the above studies, we can expect that environmentally induced phenotypic plasticity in the brain can show habitat-dependent population variation under common garden settings. Patterns emerging from common garden experiments are likely to have a genetic basis, while the habitat dependence suggests that natural selection is the driving force. However, more studies addressing geographic variation in brain plasticity, and possible population differences in the degree of plasticity, are needed to form a better view of evolutionary potential of brain plasticity itself.

## Future Directions

We have provided an overview of the published studies on intraspecific variation in brain size and architecture in the wild, and shown that there is a considerable evolutionary potential for brain divergence within species. This within-species variation provides possibilities to address evolutionary questions about brain size divergence that could not be tested with interspecific evolutionary comparative studies, or with intrapopulational plasticity studies. Unfortunately, the relatively low number of intraspecific evolutionary studies suffers from similar problems as the interspecific ones: most of them are correlative and the results are sometimes conflicting. However, considering that studying intraspecific brain size variation in the wild is an emerging field (Fig. 1), one should focus on the future possibilities rather than on the shortcomings of present and past work. By focusing on brain evolution within species, it is possible to improve our understanding of the mechanisms behind brain evolution, as both key ingredients of the evolutionary process – inheritance and selection – can be quantified and studied in detail. In fact, the array of possibilities is bewildering, but here we aim to point out two main lines of research that could lead to significant immediate progress.

The first major advance would come from applications of quantitative genetic tools on brain size variation. It is now already clear that for drawing solid evolutionary inference, data should be collected from common garden material to avoid the confusion between genetically based differences and phenotypic plasticity (Gonda et al. 46). Most of the brain evolutionary studies, both on inter- and intraspecific levels, have been based on wild caught animals of perhaps different age and/or life stages, with an implicit assumption that brain size is constant during the life of an individual. However, brain size and architecture can change seasonally, during the life of an individual or can be altered by changing environmental conditions (Pyter et al. 124; Macrini et al. 91). Environmentally induced phenotypic plasticity can often obscure the genetically based differences of a trait and might lead to false conclusions of studies based on purely wild caught samples (e.g., Alho et al. 5; Merilä 95) – an effect already demonstrated in brain variation (Gonda et al. 46). Furthermore, ontogenetic changes (e.g., Wagner 163; Lisney et al. 86; Macrini et al. 91) as well as seasonal plasticity of the brain (Nottebohm 106; Hofman and Swaab 61, 62; Tramontin and Brenowitz 159; Pyter et al. 124) can also be controlled in common garden conditions. Common garden studies, however, also offer other advantages than just ruling plasticity out. With adequate breeding designs (e.g., Falconer and Mackay 34; Lynch and Walsh 89) the different quantitative genetic components (additive genetic, maternal and environmental effects, dominance, etc.) of phenotypic variation could be disentangled both within and among populations. Further, by measuring different brain traits on the same individuals, the genetic correlations between traits could be estimated, and the competing constraint versus independent (mosaic) brain evolution hypotheses (Finlay and Darlington 35; Barton and Harvey 9) could be directly tested. Construction of the genetic variance–covariance matrix (G matrix: Lande 79) would allow estimation of the lines of least resistance (c.f. Schluter 139) and thus aid in our understanding of the constraints of brain evolution. Combining estimates of heritabilities, genetic correlations, and the G matrix with estimates of natural or sexual selection on different brain phenotypes would make a detailed reconstruction of the evolutionary process possible. Further, proper common garden material from several populations would allow us to estimate the actual quantitative genetic variation within and among populations, which, together with similar estimates of the neutral genetic variation would provide a direct test of the roles of natural selection versu*s* genetic drift behind genetically based population divergence (Merilä and Crnokrak 96; Leinonen et al. 82). Finally, and ultimately, with the current genomics tools, approaches such as genome scans (Schlötterer 138; Storz 154; Vasemägi and Primmer 161) or quantitative trait locus (QTL) mapping (Weller 168; Erickson et al. 32; Slate 145) can be used to identify the genomic regions containing the genes coding for brain variation. This line of research is particularly promising; given that already candidate gene studies (e.g., Palopoli and Patel 109) on brain size evolution have yielded exciting results (Montgomery et al. 101; Montgomery and Mundi 100). Hence, studies applying cutting-edge genomics methods could be used to test the independent versus constraint hypothesis about brain architecture evolution (see Finlay and Darlington 35; Barton and Harvey 9) directly (Hager et al. 54).

The second line of advances might result from applying the well-established, simple, and sophisticated methodology from neurobiology to the above described evolutionary framework. As the brain is an expensive tissue from the energetic point of view (Aiello and Wheeler 2), any increase in its size should be more beneficial than the cost of developing and maintaining it (e.g., Safi and Dechmann 136). However, given the many functions brain serve, linking variation in brain size to variation in any other (e.g., behavioral) traits can be difficult (Healy and Rowe 58). Further, even though the different brain parts might evolve in concert and not be entirely independent (Finlay and Darlington 35), not all changes in all brain parts might be detectable by measuring overall brain size. Studying the size of different brain parts might bring us closer to identifying functional relationships between the given neural structures and the factors that are important in their evolution. However, the functions of the main brain parts are very diverse (e.g., Kotrschal et al. 74; Striedter 155). Hence, using the volume of a part of the brain and correlating it with some, for example, behavioral trait, such as the hippocampus with food hoarding, can still be just a “proxy for more relevant and subtle changes in the structure of the brain underlying changes in behavior” (Roth et al. 133). Methods from neurobiology are available from basic histological methods to cutting-edge molecular tools. The array of neurobiological methods is bewildering, and we only aim to list a few here as examples. Basic methods include different staining methods (e.g., Nissl staining; Nissl 105) that allow one to calculate the volume of more specific brain regions within brain parts with functions defined, or calculate neuronal densities. Further, by the help of a newly developed method one can count neurons and other cell types in the brain (Herculano-Houzel and Lent 60). This provides us with a powerful tool to understand functional changes in the brain as the number of neurons might reflect the importance of a given brain structure more than its pure size (Herculano-Houzel 59). The more advanced methods consist of, for example, parallel application of different neuro-histochemical methods to visualize specific cells or components of the neurons in the brain such as antibody labeling, enzyme histochemistry, or immunofluorescence methods (Sallinen et al. 137). These latter methods/techniques have already resulted in valuable applications in easily available model systems (e.g., zebrafish, *Danio rerio*) to study very complex and important problems such as neurodegenerative human diseases (Panula et al. 110; Xi et al. 173). Such truly interdisciplinary approaches (note that the tools and knowledge are readily available for both quantitative genetics and neurobiology) would bring the understanding of both the processes and detailed function of brain evolution into reach.

## Conclusions

The enormous variation in brain size and architecture observed in nature has attracted a lot of attention in different fields of biology, including evolutionary biology. Thus far, the two main pillars of our understanding on brain variation have been macroevolutionary comparative studies of species or higher taxa and plasticity studies within populations. Interpopulation comparisons of brain size and architecture, as well as brain plasticity represent a more recent and still developing line of research in evolutionary neurobiology. This new line of research brings studies on brain size and architecture closer to mainstream evolutionary biology research where the study of spatial or geographic variation has been one of the fundaments of evolutionary investigations. The application of the outlined intraspecific evolutionary approaches should provide the basis to understand the adaptive nature of variation in brain structures as in the case of any quantitative trait. By tapping into the approaches and methods from the well-established fields of evolutionary biology and neurobiology, we envision that intraspecific studies of brain evolution can help us toward better understanding of the evolution and functional significance of variation in brain size and architecture.
